# Single-incision video-assisted thoracoscopic resection of a pedunculated solitary fibrous tumor of the pleura: case report

**DOI:** 10.1186/1477-7819-11-105

**Published:** 2013-05-22

**Authors:** Masaya Tamura, Yosuke Shimizu, Yasuo Hashizume

**Affiliations:** 1 Department of Surgery, Fukui Prefectural Hospital, Yotsui 2-8-1, Fukui 910-8526, Japan

**Keywords:** Single-incision thoracoscopic surgery, Solitary fibrous tumor of the pleura, Video-assisted thoracoscopic surgery

## Abstract

In this report, we describe the surgical resection of a pedunculated solitary fibrous tumor of the pleura (SFTP) by single-incision thoracoscopic surgery (SITS). SITS may be a suitable surgical option for pedunculated SFTPs.

## Background

Solitary fibrous tumor of the pleura (SFTP) is a relatively rare pleural tumor that generally arises from the visceral pleura as an asymptomatic pleural-based mass [1]. We present a case of SFTP, which was initially diagnosed as a thymoma during a preoperative computed tomography (CT) scan. We describe the first case of a pedunculated SFTP, resected by minimally invasive single-incision thoracoscopic surgery (SITS). Furthermore, we discuss the advantages and disadvantages of several practical treatments.

## Case presentation

**Figure 1:**
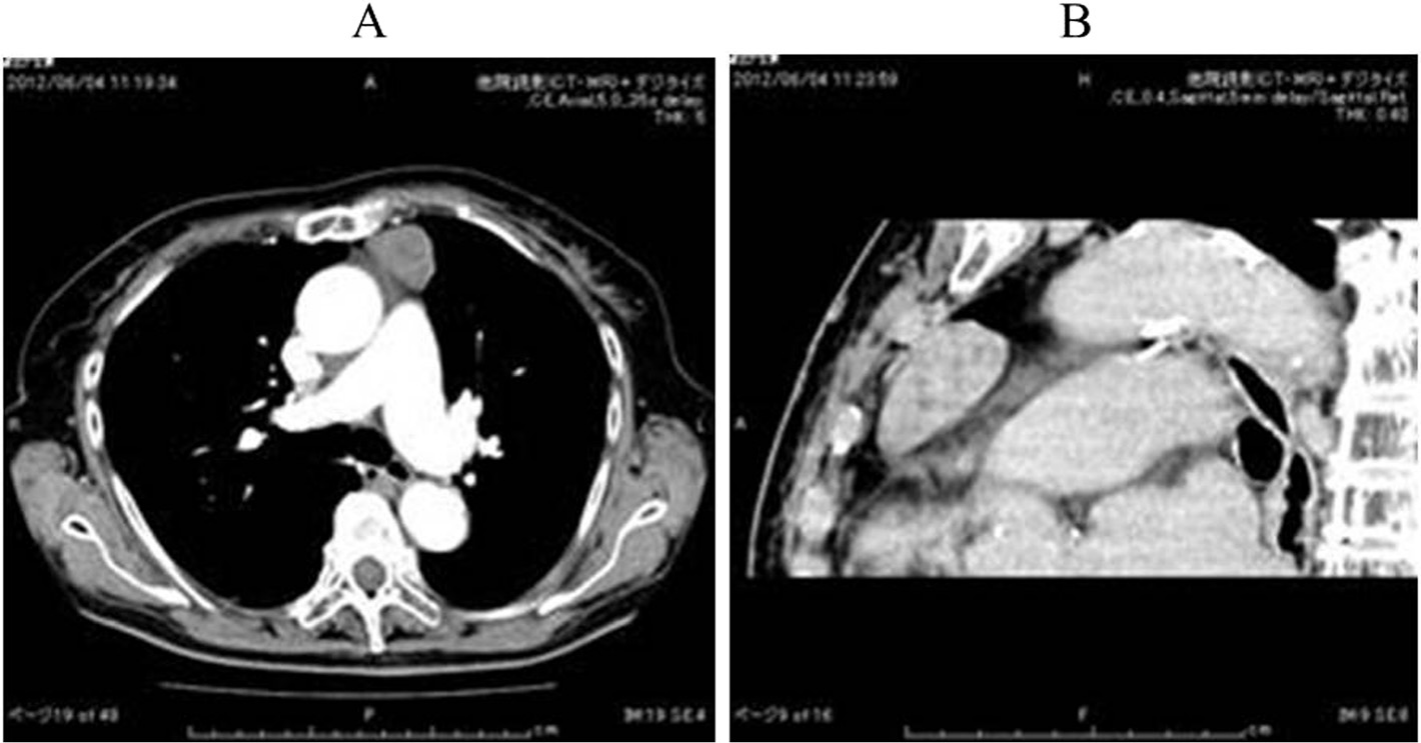
**Preoperative chest computed tomography (CT) scan demonstrated a homogenous, sharply circumscribed mass in the anterior mediastinum.** (**A**): Enhancement showed a mass that was not enhanced. (**B**) Sagittal view.

**Figure 2:**
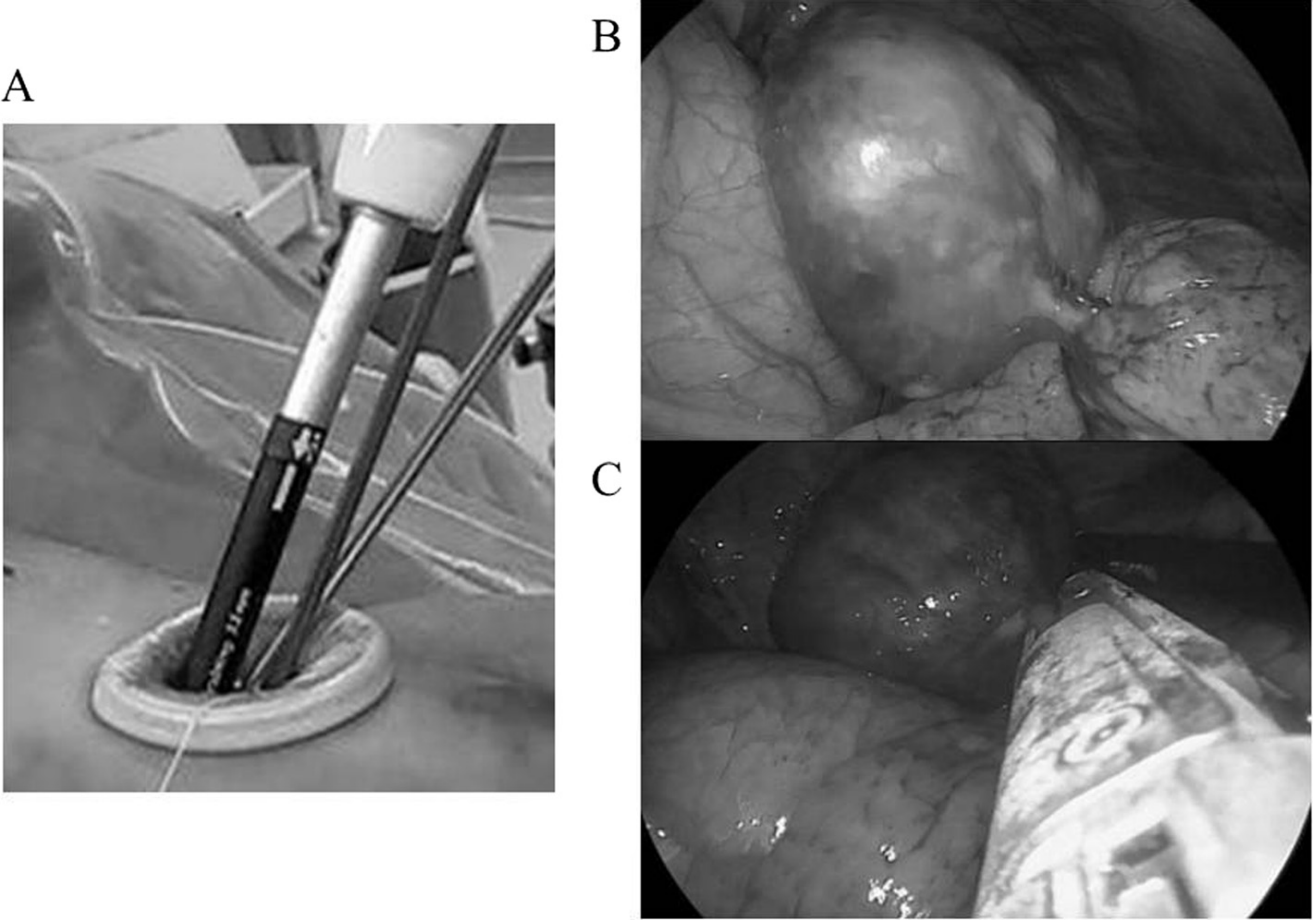
**Intraoperative findings.** (**A**) A 2.5 cm skin incision was made, and a wound retraction system (Alexis Wound Retractor, Applied Medical, Rancho Santa Margarita, CA USA) was placed through the incision. **(B**) A pedunculated tumor protruded into the thoracic cavity from the visceral pleura, and was attached to the left upper lobe. (**C**) The tumor stalk was resected using an articulating endostapler (Covidien, Norwalk, CT, USA).

An 81-year-old woman was admitted to our hospital for further examination of an abnormal shadow found on a chest CT. She had a history of thymectomy for thymoma. A CT scan demonstrated a homogenous, sharply circumscribed mass in the anterior mediastinum (Figure 1). The preoperative diagnosis was given as recurrent thymoma. However, surgery was required to arrive at a pathological diagnosis and for curative resection. The patient was given general anesthesia using one-lung ventilation and was placed in a semilateral position. Exploration through the left fifth intercostal space using a 5 mm trocar was performed. No tumor was found in the thymic tissue. However, a pedunculated tumor that protruded into the thoracic cavity from the visceral pleura was observed. The tumor was attached by its stalk to the left upper lobe, and moved freely (Figure 2B). We believed that a single-port surgery was feasible for this type of lesion.

The 5 mm incision was extended to 2.5 cm, and the wound retraction system (Alexis Wound Retractor, Applied Medical, Rancho Santa Margarita, CA USA) was then placed through the incision (Figure 2A). The tumor stalk was suspended using articulating endograspers (Covidien, Norwalk, CT, USA) and was resected using an articulating endostapler (Covidien) (Figure 2C). We did not perform frozen section during the procedure. Because the tumor was pedunculated and relatively small, it could be resected with sufficient margin.

**Figure 3:**
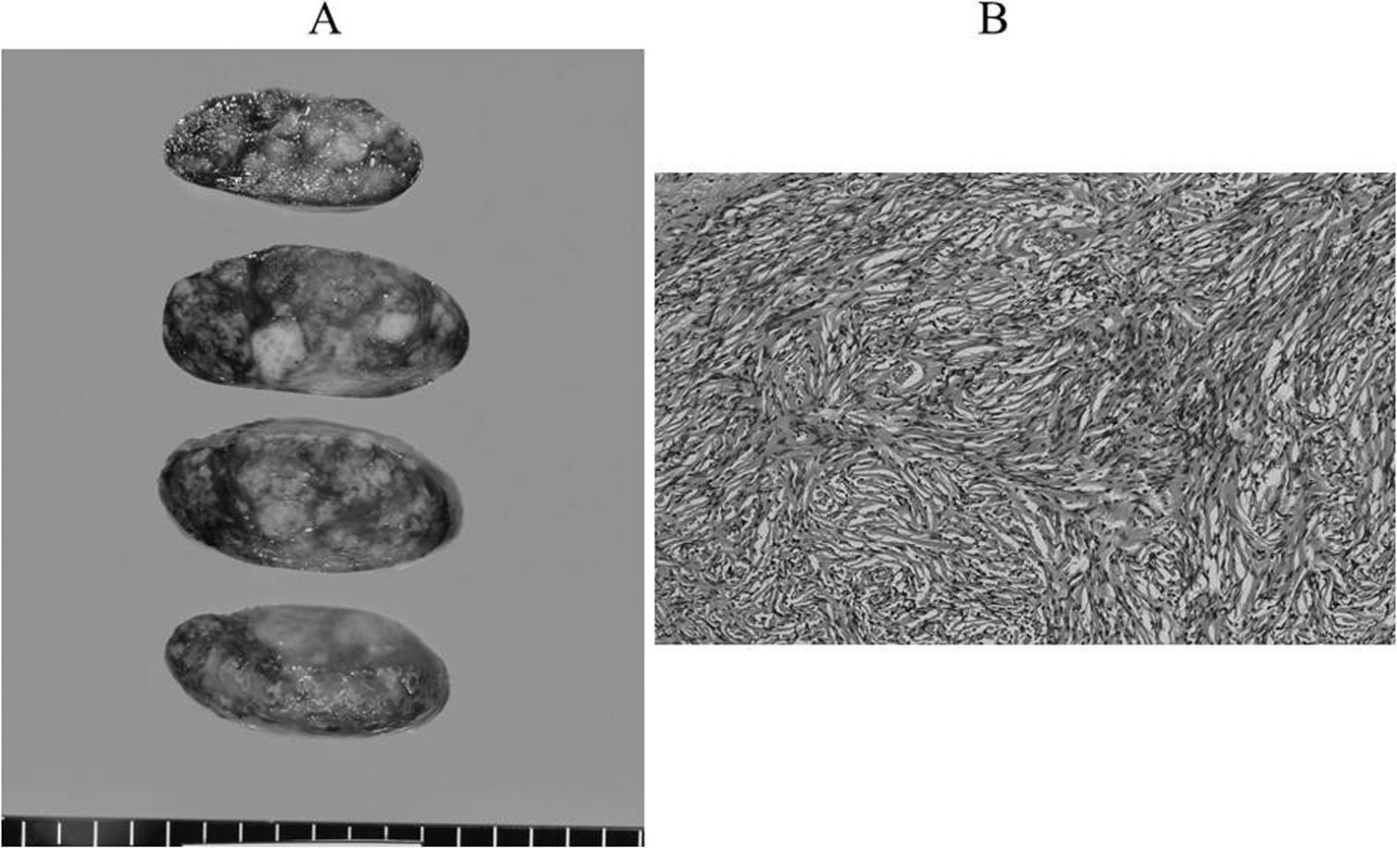
**Tumor pathology.** (**A**) Cut surface of the resected specimen demonstrated a smooth margin with solid consistency and a heterogeneous appearance (4.0 × 3. 0 × 1.8 cm). (**B**) Histological specimen demonstrated that the tumor consisted of hypocellular and moderately cellular areas of bland spindle cells in abundant collagen fibers. (H&E, ×200).

Microscopically, the tumor was characterized by spindle cells that were organized in short fascicles against a collagenous background. Areas of high cellularity and mitotic activity were absent (Figure 3). Immunohistochemical staining was strongly positive for CD34, bcl-2, and vimentin in the cytoplasm of the tumor cells. The tumor was determined to be a benign SFTP. The patient was followed up for 6 months, and no evidence of recurrence was observed.

## Discussion

Solitary fibrous tumor of the pleura is a type of mesenchymal tumor that tends to involve the pleura. Although most SFTPs are considered histologically benign, local recurrences and enlargements without any sign of invasion or metastasis have been reported [2]. Video- assisted thoracoscopic surgery is thought to be minimally invasive; therefore, it is perceived to result in less postoperative pain and faster recovery [3]. Takahama *et al*. [4] reported that VATS is a powerful and useful approach for pedunculated tumors.

Recently, VATS has become a more commonly used technique for thoracic tumor surgeries [5]. In particular, single-port VATS has been useful for specific diseases, such as pneumothorax [6]. To the best of our knowledge, there have been no previous reports on the use of single-port VATS for SFTP. Because only one intercostal space is involved, the possible advantages of SITS over conventional three-port VATS include less postoperative pain, fewer postoperative drainage days, shorter hospital stays, and cosmetic advantages. Some authors have reported less postoperative pain and less paresthesia in patients who underwent minor procedures through a single-port approach than with the classical three-port approach [7,8].

There are obvious technical problems with single-port thoracoscopic surgery. It is not a naturally ergonomic procedure, because the traditional thoracoscopic principles of triangulation are lost. In addition, positioning of multiple devices poses a problem because they are passed through a single small incision in the chest. Instruments often interfere with each other, not only within the pleural space, but also in the extrapleural space, where such attachments as a camera light lead often impede movements. To overcome these limitations, the development of new instruments is needed. Increasing the length of the camera shaft will allow an assistant to stand comfortably with his or her hands away from those of the surgeon. Use of a rotary articulating endograsper aids in achieving triangulation and assists with the use of other devices, with good results.

Nomori *et al*. [9] reported that contact metastasis and local recurrence occur at the port site. In the case presented here, the tumor was resected through a 2.5 cm incision wound, even though the tumor was 4.0 cm in diameter. A wound retraction system should provide for wound dilation and protection when a specimen is to be removed through a small incision. Initially, the patient’s tumor was thought to be a recurrent thymoma. In general, median sternotomy is planned for a thymoma. When the diagnosis is suspected preoperatively, using a single-port VATS can contribute to the avoidance of obvious excessive invasion.

## Conclusion

In conclusion, we recommend minimally invasive single-port VATS for resecting a thoracic pedunculated SFTP.

## Consent

‘Written informed consent was obtained from the patient for publication of this report and any accompanying images’.

## Abbreviations

CT: Computed tomography; H&E: Hematoxylin and eosin; SFTP: Solitary fibrous tumor of the pleura; SITS: Single-incision thoracoscopic surgery; VATS: Video- assisted thoracoscopic surgery.

## Competing interests

The authors declare that they have no competing interests.

## Authors’ contribution

M T drafted the manuscript. Y S and Y H participated in the sentence alignment and editorial work of the picture. All authors read and approved the final manuscript.
